# Tube feeding in advanced dementia: Insights from South African speech-language therapists

**DOI:** 10.4102/sajcd.v71i1.970

**Published:** 2024-02-12

**Authors:** Danette Pullen, Bhavani S. Pillay, Esedra Krüger

**Affiliations:** 1 Department of Speech-Language Pathology and Audiology, Faculty of Humanities, University of Pretoria, Pretoria, South Africa

**Keywords:** advanced dementia, feeding tube, speech-language pathologists, oropharyngeal dysphagia, palliative care, qualitative research, decision-making

## Abstract

**Background:**

Speech-language therapists (SLTs) may recommend tube feeding even with minimal research evidence of its effectiveness, and an understanding of SLTs’ perceived practices is warranted.

**Objectives:**

To qualitatively describe a sample of South African SLTs’ perceived practices regarding feeding tube placement in people with advanced dementia.

**Method:**

Semi-structured online interviews were conducted via Microsoft Teams. Eight South African SLTs with a particular interest in advanced dementia, in public and private settings, were recruited. Data were analysed using inductive reflexive thematic analysis.

**Results:**

Three main themes were identified: (1) factors influencing SLTs’ decisions for feeding tube placement in people with advanced dementia; (2) nature of clinical setting and SLTs’ decision-making and (3) SLTs’ considerations to improve management of people with advanced dementia. Existing local palliative care guidelines were not employed in decisions about tube feeding. Most participants did not recommend tube feeding during end-of-life care. Perceived burden of care influenced participants’ decisions about tube feeding.

**Conclusion:**

Speech-language therapists in South Africa likely have an increased reliance on clinical experience rather than recent research and guidelines for decisions about feeding tube placement. Findings accentuate the importance of clinical supervision, mentoring and continuous professional development in the workplace. The findings are an urgent call to action to improve SLTs’ overall practices and ethical service delivery for people with advanced dementia and their families.

**Contribution:**

Factors and needs regarding SLTs’ decision-making about feeding tubes in people with advanced dementia are highlighted.

## Introduction

Advanced dementia refers to the final stage of a progressive and incurable illness characterised by severe disability (De Jager et al., 2017; Hýden et al., 2022; Mitchell, 2015). According to Stage 7 on the Global Deterioration Scale, advanced dementia is marked, among others, by profound memory deficits, minimal verbal abilities, dependence on others for ambulation and inability to perform daily activities (Elliot et al., 2021; Hýden et al., 2022). Palliative care plays a vital role in managing people with dementia (PWD), aiming to enhance quality of life from an early stage and continuing throughout the disease progression. As the person with advanced dementia approaches the end of their life, the focus shifts to end-of-life care, emphasising comfort measures, family support and appropriate interventions for a dignified and peaceful death (Elliot et al., 2021). Eating and swallowing difficulties, including compromised mastication and an increased risk of aspiration, are common in people with advanced dementia (Espinosa-Val et al., 2020; Schwartz, 2018). Oropharyngeal dysphagia (OPD) is prevalent in 53% – 60% of people with advanced dementia with increased transition duration oral transit times being a contributing factor (Alagiakrishnan et al., 2013; Espinosa-Val et al., 2020; Guastella et al., 2022; Ijaopo & Ijaopo, 2019).

Speech-language therapists (SLTs), experts in OPD treatment, use direct and indirect treatment methods to manage patients with OPD and may recommend tube feeding (Beckley, 2017; Cloete et al., 2022; Ellison, 2016). Global controversy exists regarding the effectiveness of tube feeding and SLTs’ practices when managing people with advanced dementia (American Geriatrics Society, 2016; Cloete et al., 2022; Hýden et al., 2022; Ijaopo & Ijaopo, 2019). Despite limited evidence supporting claims that tube feeding is beneficial for PWD, South African SLTs perceived tube feeding as a standard treatment approach in PWD (Cloete et al., 2022). This perceived standard of treatment was found to be guided by clinical experience rather than research-based guidelines (Cloete et al., 2022). The mismatch between what guides decision-making may be due to a lack of local guidelines that reflect unique or extraneous factors in South Africa (Andrews & Pillay, 2017; Ashwell et al., 2020; Cloete et al., 2022; Kochovska et al., 2020; Tsao et al., 2019; Varindani Desai & Namasivayam-Macdonald, 2020). Investigating local SLTs’ practices regarding tube feeding in advanced dementia could serve as a starting point for local SLT guideline development that considers unique needs and circumstances of patients with advanced dementia. By gaining a deeper understanding of factors that influence SLTs’ decision-making, the quality of care extended to people with advanced dementia could be enhanced.

Speech-language therapists may face challenges managing people with advanced dementia due to their lack of experience in end-of-life care planning and may rely on outdated reasoning for recommending tube feeding in this population (Cloete et al., 2022; Volicer et al., 2019). Healthcare professionals require training in both palliative and end-of-life care management when managing PWD (Volicer et al., 2019). The recommended approach is to adopt a palliative care model that manages disease prognosis with support, advance care planning and pain management (Durepos et al., 2017). However, SLTs and family members may unknowingly opt for feeding tube placement to relieve perceived suffering, due to compromised nutritional intake, in people with advanced dementia, which may increase the risk of mortality (Cloete et al., 2022; Malek et al., 2018; Newman et al., 2019; Varindani Desai & Namasivayam-MacDonald, 2020). The National Institute for Health and Care Excellence (NICE) suggests tube feeding only be used for short periods during end-of-life care and supports prolonged tube feeding only when the decision is made by the patient or an advance directive (De Jager et al., 2017; NICE, 2006). The European Society for Clinical Nutrition and Metabolism (ESPEN) recommends the PWD’s representative make decisions in the absence of an advance directive, and healthcare professionals start alternative nutrition if the decision is delayed (De Jager et al., 2017). The American Geriatrics Society suggests that voluntary requests for eating and drinking by PWD should ethically override the advance directive (American Geriatrics Society et al., 2014). These factors must be considered in South Africa, where dementia care is perceived as a low priority, and palliative care is underresourced (Ashwell et al., 2020; Cloete et al., 2022; Newman et al., 2019; Volicer et al., 2019).

South Africa has limited resources; public services are only accessible to a small percentage of older people, predominantly in urban areas (Ashwell et al., 2020; Cloete et al., 2022). Private practitioners make up the majority of SLTs, experienced in working with PWD, further limiting access to specialised care (Ashwell et al., 2020; Cloete et al., 2022). The American Geriatrics Society recommends careful hand feeding as an alternative to feeding tube placement for people with advanced dementia (American Geriatrics Society et al., 2014; Chou et al., 2020; Luk et al., 2017). However, based on a recent study with South African SLTs, careful hand feeding was not reported to enhance swallowing quality or reduce eating-, drinking- and swallowing difficulties in PWD (Cloete et al., 2022; Fong, 2019; Luk et al., 2017). Comfort feeding, a management approach that involves providing nutrition through careful hand feeding, prioritises the patient’s comfort and well-being. It is primarily designed for patients with irreversible diseases, specifically those receiving palliative care, such as in the case of advanced dementia. Careful hand feeding is viewed as a specific technique rather than a broad management approach (Fong, 2019). It is crucial to investigate potential burdens and benefits of tube feeding in low- and middle-income settings like South Africa, where unqualified caregivers make use of careful hand feeding, in the context of comfort feeding (Cloete et al., 2022; Druml et al., 2016; Ijaopo & Ijaopo, 2019, Kochovska et al., 2020; Tsao et al., 2019; Varindani Desai & Namasivayam-Macdonald, 2020). These circumstances could encourage professionals’ preference for tube feeding to maintain patient well-being (Bachelor-Murphy et al., 2019; Cloete et al., 2022; Druml et al., 2016; Ijaopo & Ijaopo, 2019).

The survey study previously conducted in South Africa by Cloete et al. (2022) provides a foundation for understanding SLTs’ current practices working with PWD. However, to gain a comprehensive understanding of the topic, the current study aimed to focus specifically on advanced dementia and involves individual interviews with SLTs who have a particular interest in working with PWD.

## Methods

### Study design

The study utilised a qualitative approach with a phenomenological framework (Brink et al., 2018; Moser & Korstjens, 2018) to investigate SLTs’ perceived practices and experiences regarding feeding tube placement in people with advanced dementia. Online interviews were conducted with SLTs who had a particular interest in working with people who have dementia.

### Study population and sampling strategy

Social media platforms such as Facebook were used to advertise the study and potential participants could volunteer to participate by contacting the researcher. The advertisement was also sent to selected individuals from the researcher and supervisors’ current network base. Purposive sampling (Brink et al., 2018; Moser & Korstjens, 2018) was used and eight participants who met inclusion criteria were included: qualified SLTs, with at least 3 years of experience, and who had experience within the previous year, working with people with advanced dementia in either the private or public health sector in South Africa. Criterion sampling, a type of non-probability, purposive sampling, was used to select participants (Braun & Clarke; 2022; Brink et al., 2018). Four (50%) of the participants were considered as having expertise in working with PWD, as they either had a number of years of experience in dementia care (mean 10.75 years; range 5–25 years) or had additional qualifications. Six (75%) of the participants were actively rendering services in the private sector. Five (62.5%) participants resided in the Gauteng province (Table 1).

**TABLE 1:** Participants’ demographic information (*N* = 8).

Participant	Age (years)	SLT experience (years)	Dementia experience (years)	Dementia-related qualifications and training	Province	Healthcare setting	Clinical setting
A1	37	17	6	Rendered dementia care, as part of a multidisciplinary team, at one of two dementia clinics in South Africa; attended seminars, symposiums and short courses related to dementia	KwaZulu-Natal	Public	Acute
A2	48	26	25	Cognitive stimulation therapy; Alzheimer’s South Africa Consultant; Member of South African Healthy Ageing Association; Member of Memory care; Postgraduate research project related to dementia; FEES training	Gauteng	Private	Sub-acute, acute, frail care
A3	33	10	7	Master’s degree: AAC for adults with receptive difficulties; various CPD events related to palliation and tube feeding; FEES training	Eastern Cape	Private	Acute
A4	42	18	4	LSVT Loud training; VitalStim qualified; FEES training	Gauteng	Private	Sub-acute
A5	31	10	5	Completed multiple courses via the Wicking Dementia Centre; FEES training	Gauteng	Private	Rehabilitation centre
A6	27	4	4	LSVT Loud training; attended South African geriatric society continuous professional development series – dysphagia in the geriatric population; presented to various frail care facilities: dysphagia in the geriatric population	Gauteng	Private	Acute
A7	44	23	13	Attended multiple seminars and symposiums related to dementia care	Eastern Cape	Private	Acute, rehabilitation centre
A8	26	4	3	Adult dysphagia course; adult neuro skills building – multidisciplinary team initiative	Gauteng	Public	Acute

SLT, speech-language therapist; FEES, fiberoptic endoscopic evaluation of swallowing; LSVT, Lee Silverman voice treatment.

### Data collection

Data were collected over a period of 6 months via online interviews on a video conferencing platform, Microsoft Teams. Once-off online interviews created an opportunity for accessibility to various participants irrespective of their geographical location or time constraints (Moser & Korstjens, 2018). The interviews were guided by a self-compiled semi-structured interview schedule. The questions were tailored according to relevant literature and a recent quantitative study in the same context (American Geriatrics Society et al., 2014; Ashwell et al., 2020; Baijens et al., 2016; Beckly, 2017; Campbell et al., 2011; Car et al., 2017; Cloete et al., 2022; Colette et al., 2022; Desai & Namasivavam-Macdonald, 2020; Ellison, 2016; Hickey & Bourgeois, 2017). Kuven, 2018; Luk et al., 2017; Malek et al., 2018; Meier & Ong, 2015; Newman et al., 2019; Punchik et al., 2018; Schwartz et al., 2014; Volicer et al., 2019; Volkert et al., 2015; Vose et al., 2018; Wright et al., 2019).

### Data analysis

The study used verbatim transcription of data, which was uploaded for inductive coding and reflexive thematic analysis using ATLAS.ti, a qualitative data analysis and research software. The transcripts were reviewed multiple times to organise the data into appropriate codes, which were then grouped and developed into themes. To increase the reliability and validity of the collected data, the plausible themes were discussed with the second and third authors. The study followed the guidelines of Braun and Clarke (2022) and Terry et al. (2017).

### Ethical considerations

The study received institutional ethical clearance from the Faculty of Humanities’ Research Ethics Committee at the University of Pretoria on 09 December 2021 (protocol number: HUM022/1021). Participation was voluntary and written informed consent was obtained from all participants.

## Results

The study identified three main themes and multiple sub-themes that were in line with its aim. Table 2 provides an overview of the themes and sub-themes related to participants’ practices regarding feeding tube placement in people with advanced dementia.

**TABLE 2:** Themes and sub-themes identified in the study.

Themes	Sub-themes
Factors influencing SLTs’ decisions for feeding tube placement in people with advanced dementia	Intrinsic factors
	SLTs’ level of experience
	SLTs’ morals and beliefs
	Awareness of guidelines
	Extrinsic factors
	Availability of local guidelines
	Advance directives
	Cultural and religious background of families
	Perceived family caregiver burden
Nature of clinical setting and SLTs’ decision-making	Clinical setting
	Acute setting
	Instrumental assessments
	Feeding tube consideration and duration
	SLTs’ role
	Compensatory strategies
	Perceived nursing caregiver burden
	South African challenges
	Socioeconomic status
	Geographical location
	Limited healthcare resources
SLTs’ considerations to improve the management of people with advanced dementia	Early involvement of SLTs Continuous mentorship and collaborative practice among healthcare professionals

SLTs, speech-language therapists.

### Factors influencing speech-language therapists’ decisions for feeding tube placement in people with advanced dementia

The theme of decision-making highlights intrinsic and extrinsic factors that influence SLTs’ decisions regarding feeding tube placement in people with advanced dementia. Participants believed that increased experience and exposure, coupled with evidence-based practice, resulted in improved decision-making. Two participants expressed that their experience had served them best when dealing with OPD in people with advanced dementia:

‘I enjoy implementing whatever I read and try new things out, but for now it’s just kind of from experience and what has served me the best so far.’ (A4)

‘It is something that takes a couple of years and a couple of different patients and different families to start making sense and putting the picture together.’ (A5)

To be deemed competent in providing services for individuals with dementia, SLTs must possess familiarity with appropriate protocols and guidelines (American Speech-Language-Hearing Association [ASHA], 2016; HPCSA, 2019). Five SLTs in this study referred to guidelines from organisations such as the American Speech-Language-Hearing Association, the ESPEN and the American Geriatric Society related to feeding difficulties in people with advanced dementia. However, one participant held the view that there is limited research on the benefits of feeding tubes, stating:

‘… in terms of the pros, that I’ve been reading up on a little bit as well … own bias, really trying to get both sides and with the right guidelines, with the right intervention and follow up, it [*feeding tube placement*] can be a good thing.’ (A3)

Seven participants appeared to be unaware of local guidelines including information related to feeding tube placement in people with advanced dementia, such as the Health Professions Council of South Africa’s (HPCSA) 2019 ethical guidelines on palliative care, the National Policy Framework and Strategy for Palliative Care (2017) and the Standards for Palliative Care in South Africa (2017). Three participants believed that there are limited local guidelines related to feeding tube placement in the advanced dementia population. It is possible that SLTs may be using international guidelines due to a lack of awareness of local guidelines.

Participants’ beliefs also appeared to influence decision-making. Specifically, participants expressed their opinion that feeding tube placement is associated with prolonging life and how this may be ethically and morally questionable:

‘You say, “Oh, don’t worry, there’ll be no stress for eating. You just put the food in this tube and it’s lovely. You never have to stress about it again”, and families who have been “sukkel-ing” [*slang for having difficulty with*] and carrying on trying to get the food in think “oh, this is the answer to my prayers”, you know, but we are changing the course of people’s lives and I think we don’t take enough of a conscious calculated ethical or moral decision making process around it.’ (A2)

‘… it is kind of until they pass away and that’s more where the ethical part of it comes in. Because now you are kind of keeping someone alive but sending them home to die.’ (A3)

Five participants expressed the belief that feeding tube placements do not improve quality of life for people with advanced dementia:

‘They [*SLTs*] have good intentions, because they have realised that oral intake is not sufficient anymore, but they’re causing harm, because the life is sustained artificially. There is just so many variables that in my perspective do not guarantee a great quality of life going forward.’ (A5)

Two participants held the belief that feeding tubes can increase quality of life by ensuring adequate nutritional intake:

‘Now, they can feed better, they look healthier, they are less frequently sick. If they do not, they are back and forth every two months. With better feeding [Percutaneous Endocscopic Gastrostomy (PEG) feeding], there is definitely better health.’ (A7)

Participants’ decision-making appeared to be influenced by their own cultural and religious backgrounds, which they may not have been fully aware of. Three participants claimed that their culture or religion did not affect their decisions regarding feeding tube placement in people with advanced dementia, and that they relied solely on clinical experience:

‘I try to approach things clinically and with as much evidence to support decisions as much as possible.’ (A5)

‘It’s one of those things that might not be a popular opinion, but unfortunately within the medical realm, there is not really place for religion and culture to affect your decisions and it is right, because we are working with medicine and it is scientific and there is a right and there is a wrong.’ (A6)

Two participants shared views on how their religion influenced their decisions. Participant A4 felt that her religion was an important factor but as she gained clinical experience, her decisions were increasingly guided by her experience and skills:

‘Initially, when I just started working in this field, my religion, in a very youthful sense, played a big role. I felt we are not to decide whether a patient lives or dies. My view on feeding and my knowledge and experience has changed. Also, making peace with death and the process of death. So, I think it’s more experience that plays a role, than my religion.’ (A4)

Participant A8 expressed how her religious beliefs influence her recommendations to ensure nutritional health in people with advanced dementia:

‘In terms of religion, we believe that you need to look after your health, holistically. If it means you going to a doctor …you allow yourself medical care purely because your religion requires that you look after your health. I will put it in [*feeding tube*], purely because I feel it can help, but obviously only through God’s help, can it help.’ (A8)

Two participants disclosed that cultural stereotyping could impact their interactions with patients and recommendations:

‘I also acknowledge the fact that I’m dealing with an Indian family, and this is my expectations … I’m dealing with an African family, so this is the likely thing that I would encounter and if I’m dealing with a typical Afrikaans family then this is the kind of situation I might encounter. Maybe it’s also my expectation on the details that affect how I see that.’ (A7)

‘We always have cultural bias, and you are trying to be so aware of your own bias because it can slip in so much.’ (A3)

Participants acknowledged that the final decision related to feeding tube placement lies with a patient’s family. Seven participants emphasised the importance of providing informational counselling to families prior to making decisions. Participants also expressed the belief that families’ cultural and religious backgrounds could influence their attitudes towards advanced dementia and tube feeding. Six participants noted that cultural factors such as the influence of traditional healers or belief that the person with advanced dementia is bewitched may lead to a decision against feeding tube placement. Overall, participants perceived that culture has a bigger influence than the family’s religious orientation:

‘I remember there was one particular family that we had, and they were convinced that their father was bewitched. And that was why he was presenting in this manner.’ (A1)

‘You have to let the patient sign to that – to say that “I want to take my loved one out of the hospital to go and do this [*go to a traditional healer*] and I am basically refusing care from the hospital.”’ (A8)

The presence of an advance directive was found to facilitate collaboration with family members, as reported by six participants. They felt that advance directives eased decisions related to feeding tube placement. However, two participants mentioned that advance directives primarily relate to palliative considerations such as ventilation and resuscitation, rather than feeding tube placement.

Six participants felt feeding tube placement should not be recommended during palliative care for people with advanced dementia, citing reasons such as decreased energy levels, poor cognitive abilities and increased risk of infection from dislodged feeding tubes. In contrast, some attempts to alleviate perceived caregiver burden may lead to percutaneous endoscopic gastrostomy (PEG) tube placement in this population:

‘… maybe there is not a good plan in place, or the family has not figured out a frail care setting or there is not consensus about who is going to take care of this member [*the patient with advanced dementia*]. Then I really see that PEG placements happen very quickly.’ (A3)

Three participants reiterated that feeding tube placement can ease perceived caregiver burden, while two participants disagreed and expressed the view that PEG tube placement increases caregiver burden:

‘It is decreasing their mortality, because obviously they get longer nutrition, their bodies are healthy, …have a little bit more strength. It is just that their mind is not there. So, you are decreasing mortality, yes, but like I said – you do not know how long the family is going to continue doing it [*feeding through PEG tube*], because it is such a big responsibility on them.’ (A8)

### Nature of clinical setting and speech-language therapists’ decision-making

This theme highlights the role of the clinical setting and geographical location in South Africa in shaping SLTs’ practices regarding feeding tube placement in people with advanced dementia. The findings suggest that the acute care setting is the most prevalent setting where participants encountered people with advanced dementia, and six participants reported their likelihood to recommend feeding tubes if patients are admitted to acute care settings.

Five participants expressed their need for access to instrumental assessments, such as videofluoroscopic swallowing study (VFSS) and fiberoptic endoscopic evaluation of swallowing (FEES), to assess OPD and facilitate improved decision-making. Participant A5 emphasised that instrumental assessments, particularly FEES, could enhance the effectiveness of informational counselling by providing tangible evidence in addition to clinical opinion. This sentiment was echoed by two other participants:

‘If objective assessments would be more accessible, then I would know I am making the right decisions and maybe I would have more evidence to educate the patients and families, because “look, you can see it on the screen, what’s happening”.’ (A5)

Two other participants felt that patients with advanced dementia often present with poor health and cognition affecting accessibility to, and the feasibility of, instrumental assessments. Five participants indicated that nasogastric tube (NGT) placements serve as a temporary solution, not exceeding 6 weeks. After 6 weeks, a permanent decision should be made: transitioning to oral feeds or PEG tube placement, especially for discharge to home. Participants felt that NGT feeding poses a greater risk for aspiration as it can dislodge easily, and it requires frequent follow-up appointments.

Some SLTs (*n* = 3) considered PEG tubes as a safer option. Participants noted that certain rehabilitation and frail care facilities admitted patients under two conditions, namely feeding orally or feeding via PEG tubes, as an attempt to decrease the perceived burden of care on nursing staff. Discharge planning is believed to be further complicated by socioeconomic factors. Four participants felt that PEG tube placement, in people with advanced dementia, living in a rural setting with limited resources, is not feasible. Participants expressed that poor access to basic sanitary resources may increase the risk of aspiration. The person with advanced dementia’s health was perceived to be further exacerbated by poverty, affecting optimal nutritional intake:

‘… it is often times that someone maybe lives very rurally. They do not have access to clean water, so they are not going to be able to clean the PEG tube nicely, or they do not have the equipment to actually blend the feeds to pass it through the PEG tube …’ (A6)

Alternatively, participant A7 considered socioeconomic challenges and how it affects access to healthcare. Participants believed that the progressive nature of advanced dementia leads to poor adherence of follow-up appointments. This is exacerbated by contextual factors such as poor access to healthcare due to increased travelling distance and decreased transport availability, further aggravated by financial constraints. For these patients, who also present with ongoing feeding difficulties, the participant would consider PEG tube placement:

‘It also depends on whether the patient will be able to return for follow ups as often as they should. If they are from really far away then you have to consider [*most probably pensioners*] that they cannot afford to keep coming back for review, so my decision is also linked to that [*the distance*]. Then that has to be a quick decision and a more permanent one and most convenient.’ (A7)

Participant A1 reiterated this view as there are few dementia clinics in South Africa. Despite these challenges, five participants verbalised that feeding tube placement is considered a team decision and should be a collaborative practice:

‘I am very lucky to work in a multidisciplinary team. We discuss it with the geriatrician or the physician, the pulmonologist or the neurologist if need be. It is very much a team decision. It is never down to just my decision, and obviously consulting with the families as well.’ (A2)

All participants (*N* = 8) indicated that the SLT’s role in the clinical team is to advocate for the patient’s and family’s rights and to provide informational counselling. Contrastingly, three participants mentioned that they would still make recommendations related to feeding tube placement, despite the patient’s wishes indicated in the advance directive:

‘As from a clinical perspective, I’m still saying that that is my recommendation, but however, the family or the person with dementia has stipulated in whatever it may be [*advance directive*].’ (A8)

‘My recommendations will kind of still stay the same in terms of these are the pros and cons of both.’ (A3)

To ensure safe oral feeding in people with advanced dementia, who also present with OPD, all participants revealed a preference towards compensatory strategies such as careful hand feeding, environmental modifications such as modifying diets by following international dysphagia diet standardisation initiative guidelines (IDDSI), and the use of the Frazier free water protocol (Cichero et al., 2017; Panther, 2005).

Two participants expressed that understaffed units may lead to poor adherence to compensatory feeding strategies and an increase in NGT placements within the acute setting, to relieve the perceived healthcare burden experienced by nurses. This was particularly emphasised when related to public acute healthcare settings:

‘We are not an ideal gold standard where it is a nurse to a patient. It is sometimes one nurse to six patients … ten patients. She has to now feed all these patients and by the time she is done, it is already the next feed (for example). There are other responsibilities – medication, looking at their vitals… That is also our safety net – to know that the NGT is in, because sister is not going to feed that full bowl of food.’ (A8)

### Speech-language therapists’ considerations to improve the management of people with advanced dementia

Four participants highlighted early involvement of SLTs during management of people with advanced dementia. This may lead to timely informational counselling, allowing the SLT to guide families when making important decisions, especially during palliative care. Additionally, earlier initiation of treatment may delay the progression of the disease:

‘If we catch it [*dementia*] earlier, then we can delay the whole thing, obviously the governing bodies such as SASLHA, Clinical practice guidelines that define the palliative care approach and end-of-life care management advanced stages, it is going to come, but it is delayed for as long as possible.’ (A1)

‘Whether it be me or someone else, but hopefully a speech therapist, to just say what we are expecting, what the prognosis is, so that it does not lead to a rash decision when they come into acute with late stage [*advanced dementia*], but that the family is already aware of the options from day one and that the actual person with dementia can have a say in how they would like their quality of life to be at the end stages.’ (A3)

Two participants highlighted the importance of continuous mentorship and collaboration between healthcare professionals to render optimal services to people with advanced dementia:

‘I was initially challenged by a doctor that I worked with in a sub-acute unit, where I felt very strongly that this man should get an NGT and eventually a PEG tube and the doctor said “What for? What do you think you’re going to gain for this person?” After a lot of years of working together, you kind of came to a middle ground, but from my personal experience, it made me start thinking in another way.’ (A4)

‘I strongly feel the need for mentorship in this specific area of practice with speech.’ (A6)

## Discussion

The study revealed that SLTs’ decisions regarding feeding tube placement in people with advanced dementia were influenced by a number of factors. Participants relied heavily on their clinical experience rather than available literature. Participants’ work setting and geographical location may alter decisions on whether patients are recommended feeding tubes. Participants revealed that SLTs’ perspectives on culture and religion could change their decisions.

**FIGURE 1:**
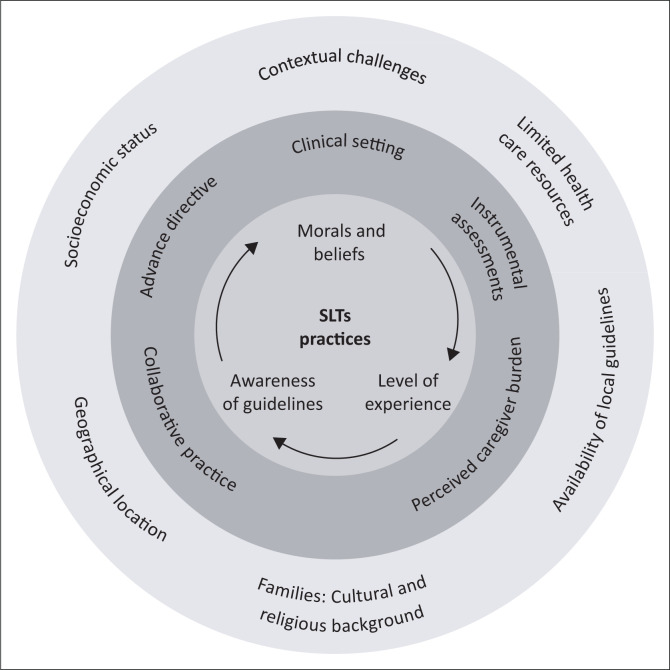
Speech-language therapists’ practices and influencing factors related to decisions about tube feeding in the advanced dementia population.

Figure 1 depicts the feeding tube placement decision-making process of SLTs in patients with advanced dementia who have difficulty with eating, drinking and swallowing and the various factors that can influence their recommendations.

An ecological approach (Bronfenbrenner, 1979) is followed (Figure 1) to illustrate the complex interplay between SLTs and various environmental factors that may impact decision-making when treating individuals with advanced dementia. This approach acknowledges that SLTs are not solely responsible for their decisions, as external factors can also influence their choices. Intrinsic factors, such as SLTs’ personal beliefs, extrinsic factors such as guidelines, and contextual challenges like socioeconomic status and geographical location, are included. The interplay between these factors and their impact on SLTs’ approaches to treatment is depicted. Limited resources and gaps in knowledge of current available evidence may lead to consideration of feeding tube placement, while personal beliefs and positive experiences with encouraging eating and drinking may favour palliative care and careful hand feeding, in the context of comfort feeding. Understanding these factors is vital for providing additional support to SLTs to provide appropriate person-centred care to people with advanced dementia.

Existing local guidelines on palliative care were not mentioned by SLTs, which likely contributes to a mismatch between decision-making and best practice. The lack of awareness of guidelines, combined with the absence of SLT clinical practice guidelines, reflects the unique challenges present in South Africa (Andrews & Pillay, 2017; Ashwell et al., 2020; Cloete et al., 2022; Kochovska et al., 2020; Varindani Desai & Namasivayam-Macdonald, 2020; Tsao et al., 2019), which may mean that SLTs rely more often on clinical experience rather than recent literature. A need for contextually relevant guidelines for SLTs developed by local governing bodies and/or professional associations is highlighted to support SLTs in dementia care and address challenges specific to South Africa.

The SLTs in the study demonstrated partial alignment with the palliative care approach prioritising home-based visits by SLTs (Drenth et al., 2018) by acknowledging that tube feeding does not improve quality of life of people with advanced dementia, but some participants still believed it could support optimal nutrition. This contradicts current evidence that neither quality of life nor nutrition is enhanced by tube feeding in such patients (American Geriatrics Society, 2016; Hýden et al., 2022; Ijaopo & Ijaopo, 2019; Payne & Morley, 2018). Additionally, participants appeared to make decisions influenced by their own lived experiences, such as cultural stereotyping and imparting moral beliefs, which may have compromised the quality of life of their patients with advanced dementia. Healthcare professionals tend to make decisions based on emotions rather than evidence-based findings (Kozlowski et al., 2017). Although cultural sensitivity is important in a diverse context (Mash, 2016; Stellenberg & De Wet, 2016), it may introduce personal bias into recommendations that do not align with literature. To ensure that the care of individuals with advanced dementia prioritises quality of life over longevity (Drenth et al., 2018), it is essential for SLTs to be cognisant of how their own cultural and religious beliefs, as well as those of the patient’s family, may influence their decision-making. Speech-language therapists should be educated about current evidence-based research and culturally sensitive practices related to people with advanced dementia at university to prevent personal biases from influencing decision-making (Beldhuis et al., 2021).

In palliative illnesses such as advanced dementia, end-of-life care is inevitable, and continuum of care is crucial (Durepos et al., 2017). Participants preferred compensatory strategies, that is, careful hand feeding, to promote exclusive oral feeding and improve quality of life before considering enteral feeding, in line with current research (American Geriatrics Society et al., 2014; Chou et al., 2020; Luk et al., 2017). In South Africa, the presence of contextual challenges, including caregiver burden, financial expenses associated with travel to healthcare facilities for follow-up appointments, and time constraints, can complicate the continuum of care. As a result, SLTs may suggest PEG tube placement as a potential solution. However, PEG tube placement does not necessarily offer a cost- and time-effective solution. Nursing home residents with advanced dementia receiving tube feeding often require hospitalisation due to feeding tube complications, despite the caregiver burden being lessened (Hwang et al., 2014; Yuen et al., 2022). Contextual constraints place a clinical burden on SLTs that inevitably compromise the management of patients with advanced dementia. To better support SLTs in making evidence-based decisions, measures such as prioritising home-based visits should be considered to ensure optimal continuity of care (Drenth et al., 2018). Furthermore, healthcare settings should prioritise professional development regarding palliative care and end-of-life care management (Cloete et al., 2022; Huang et al., 2020). This may assist SLTs in prioritising the quality of life of patients by delivering personalised care within their familiar surroundings, alongside family counselling and training to maintain optimal continuity of care, despite contextual challenges (Drenth et al., 2018).

Participants mentioned that access to instrumental assessments could improve information-giving to families when considering tube feeding in people with advanced dementia who present with OPD. Frailty of patients was identified as a clinical obstacle affecting access to such assessments (Dziewas et al., 2017). Participants highlighted the applicability of FEES to the advanced dementia population, where cognitive decline may limit the feasibility of other assessments, citing benefits such as portability and continuity of care (Dziewas et al., 2017; Langmore et al., 2007; Warnicke et al., 2010, 2016; Wirth et al., 2016). Instrumental assessments, such as FEES, could play a vital role in comprehensively assessing swallowing difficulties, detecting aspiration and determining appropriate management for individuals with advanced dementia (Payne & Morley, 2018). However, limited access to instrumentation, few opportunities for local training and the logistics of an elderly patient being submitted to an invasive procedure may hinder the practicality of implementing FEES in South Africa with PWD. Further research could explore the effectiveness of alternative, accessible and cost-effective approaches like telemedicine and mobile health technologies in facilitating remote access to instrumental assessments and providing training for healthcare professionals in resource-limited settings. Additionally, efforts should be directed towards improving clinical training in dysphagia to reduce the reliance on expensive instrumental investigations, particularly in resource-limited settings.

Participants believed their role was to advocate for patients’ rights, but some made recommendations that contradicted patients’ advance directives. This deviation from patient autonomy goes against principles of palliative care, as noted in previous research (Durepos et al., 2017; Huang et al., 2020). The study suggests that although SLTs demonstrated a basic understanding of ethical considerations in palliative care, there are concerns regarding their limited efforts to seek input from peers or supervisors, consider research evidence and engage effectively with patient and family preferences. The lack of comprehensive engagement raises questions about their decision-making process, suggesting their actions are likely driven by limited knowledge rather than intentional disregard for ethical considerations. It is crucial to prioritise preferences of individuals with advanced dementia and their families when making treatment decisions. Care goals should be established collaboratively, and the best available evidence discourages tube feeding while promoting careful hand feeding or comfort feeding (Mitchell, 2015). The study highlights the need for healthcare settings in South Africa to regularly review and enforce ethical guidelines. By adhering to the HPCSA ethical code of conduct, healthcare professionals, including SLTs, can promote patient autonomy and uphold ethical standards in palliative care (HPCSA, 2016).

The acute setting was identified as the context where participants often opted for tube feeding usually due to dehydration, as a result of reduced oral intake, OPD and/or aspiration pneumonia. In this study, participants stated that PEG tube insertion would be considered if the person with advanced dementia had not established oral feeds after 6 weeks or if the NGT needed to be removed upon discharge and the swallowing and/or eating difficulties persisted. Contrary to the reasons that influenced participants to opt for tube feeding, literature states that in cases of advanced dementia, enteral feeding, NGT or PEG tube, has not been found to improve nutritional status, prevent aspiration pneumonia or extend survival. Prolonged tube feeding in advanced dementia may lead to increased discomfort, agitation and risk of infection in patients (Volkert et al., 2015). This study highlighted a knowledge gap as SLTs did not consider tube feeding placement during the palliative phase of dementia, despite dementia being considered a palliative illness from its onset (Drenth et al., 2018). This finding indicates a lack of understanding and the connection between the concepts of palliative and end-of-life care related to advanced dementia. This finding is troubling, given that ESPEN guidelines only recommend tube feeding for mild-to-moderate cases of dementia, and prolonged tube feeding in advanced dementia should only be considered if informed by the patient or an advance directive (De Jager et al., 2017; NICE, 2006). The importance of education of SLTs regarding tube feeding use in advanced dementia is reiterated. It is crucial that healthcare professionals, including SLTs, understand the palliative nature of dementia and the appropriate use of tube feeding in advanced cases, particularly the use of tube feeding in advanced dementia. Acquiring a strong foundation in ethical practice, palliative care and end-of-life care management through undergraduate training and continuous professional development may enable SLTs to make informed decisions based on patient needs, preferences, ethics and evidence-based guidelines. The value of clinical supervision by experienced SLTs and mentoring in the workplace for qualified SLTs is also emphasised.

Decision-making about feeding tube placement for people with advanced dementia can be challenging and complex for SLTs in low-resourced settings. However, the study highlighted that such decisions should be a collaborative practice among a team of specialised healthcare professionals using recent evidence available. This could be achieved through early multi-disciplinary team (MDT) involvement, collaboration in end-of-life discussions, and establishing discharge and care planning alongside family and caregivers with the view of care having a palliative focus (Anantapong et al., 2022; Cloete et al., 2022; Mitchell, 2015; Toles, 2018). To ensure best practice in the management of advanced dementia, it is crucial to provide continued mentorship to SLTs and encourage collaborative discussions among allied healthcare professionals (Anantapong et al., 2022; Cloete et al., 2022). To explore partially understood topics, SLTs could use designated discussion forums or groups focused on special interests, highlighting the importance of continuing professional development and continued research efforts.

Further research is needed to improve decision-making for feeding tube placement in people with advanced dementia in South Africa. Large-scale studies to explore factors that impact decision-making among healthcare professionals, as well as assessing the roles of other team members, and exploring ethical and legal dilemmas, are warranted. Such research can ensure ethical and enhanced quality of care to prioritise patient autonomy and involve caregivers for improved decision-making.

## Conclusion

Speech-language therapists in South Africa likely have an increased reliance on their own clinical experience rather than incorporating recent evidence and published guidelines when making decisions about feeding tube placement for people with advanced dementia. Speech-language therapists’ need for appropriate knowledge on the value of tube feeding was highlighted indirectly. Updated local guidelines and continued professional development in the form of education and training for qualified SLTs are required and may improve palliative and end-of-life care for people with advanced dementia. Interprofessional collaboration among healthcare professionals and aligning goals of care with appropriate treatment options are important to ensure best practice and ethical decision-making throughout the continuum of care for the advanced dementia population. It is concerning that many SLTs are not basing decisions about people with advanced dementia on recent research. However, it is still important to consider clinical wisdom and client values as two important components of evidence-based practice (Baum-Baicker, 2017). The study highlights a caveat in clinical supervision and mentoring to build capacity in qualified SLTs. The findings are an urgent call to action to improve SLTs’ overall practices that influence patient and family’s care and quality of life. Continuous professional development, not only to improve clinical decision-making but also to facilitate sound ethical practices in the workplace for already-qualified SLTs working with PWD, may be a starting point.

### Strengths and limitations

The study included only a small sample of SLTs with a particular interest in managing people with advanced dementia. Therefore, findings cannot be generalised, but still provide useful insights into the decision-making and knowledge needs of South African SLTs working with people with advanced dementia.

## References

[CIT0001] Alagiakrishnan, K., Bhanji, R.A., Kurian, M., & Naugler, C. (2013). Oropharyngeal dysphagia in older adults. A review. *World Journal of Gastroenterology*, 19(28), 4649–4663. 10.3748/wjg.v19.i28.4649

[CIT0002] American Geriatrics Society. (2016). PEG tubes and placement in advanced dementia. *Journal of the American Geriatrics Society*, 64(2), 365–368. 10.1111/jgs.13902 26783046

[CIT0003] American Geriatrics Society Ethics, C., Clinical, P., & Models of Care, C. (2014). American Geriatrics Society feeding tubes in advanced dementia position statement. *Journal of the American Geriatrics Society*, 62(8), 1590–1593. 10.1111/jgs.12924 25039796

[CIT0004] American Speech-Language-Hearing Association. (2016). *Scope of practice in speech-language pathology [Scope of practice]*. Retrieved from https://www.asha.org/policy/sp2016-00343/

[CIT0005] Anantapong, K., Davies, N., & Sampson, E.L. (2022). Communication between the multidisciplinary team and families regarding nutrition and hydration for people with severe dementia in acute hospitals: A qualitative study. *Age and Ageing*, 51(11), afac230. 10.1093/ageing/afac230 36434801 PMC9701106

[CIT0006] Andrews, M., & Pillay, M. (2017). Poor consistency in evaluating South African adults with neurogenic dysphagia. *South African Journal of Communication Disorders. Die Suid-Afrikaanse Tydskrif vir Kommunikasieafwykings*, 64(1), e1–e14. 10.4102/sajcd.v64i1.158 PMC5842977 28155280

[CIT0007] Ashwell, A., Jacobs, R., Docrat, S., & Schneider, M. (2020). *The impact of COVID-19 on long-term care facilities in South Africa with a specific focus on dementia care*. Retrieved from http://sifar.org.za/sites/default/files/field/file/SA%20LTCCovid%201st%20interim%20report_10%20July%202020_Appendix%20added.pdf

[CIT0008] Bachelor-Murphy, K., Barroso, J., & Volicer, L. (2019). Feeding tubes in advanced dementia: Knowledge and practice of speech-language pathologists. *American Journal of Speech-Language Pathology*, 28(3), 1196–1208. 10.1044/2019_AJSLP-18-0239

[CIT0009] Baijens, L.W.J., Clavé, P., Cras, P., Ekberg, O., Forster, A., Gerald, F., & Leners, J.-c. (2016). European society for swallowing disorders – European union geriatric medicine society white paper: Oropharyngeal dysphagia as a geriatric syndrome. *Clinical Interventions in Aging*, 2016, 1403–1428. 10.2147/CIA.S107750 PMC5063605 27785002

[CIT0010] Baum-Baicker, C. (2017). Defining clinical wisdom. *Journal for the Advancement of Scientific Psychoanalytic Empirical Research*, 1(2), 71–83. Retrieved from https://www.researchgate.net/profile/Cynthia-Baum-Baicker/publication/325367001_Defining_Clinical_Wisdom_Jrl_for_the_Advancement_of_Scientific_Psychoanalytic_Empirical_Research_JASPER_International_12_71-83/links/5b0818450f7e9b1ed7f2dff7/Defining-Clinical-Wisdom-Jrl-for-the-Advancement-of-Scientific-Psychoanalytic-Empirical-Research-JASPER-International-12-71-83.pdf.

[CIT0011] Beckley, E.T. (2017). Patient wishes before risk: How do we honor patients’ decisions about their swallowing treatment when they have dementia-and there’s aspiration risk?. *The ASHA Leader*, 22(5), 40–47. 10.1044/leader.FTR1.22052017.40

[CIT0012] Beldhuis, I.E., Marapin, R.S., Jiang, Y.Y., De Souza, N.F.S., Georgiou, A., Kaufmann, T., Forte, J.C., & Van der Horst, I.C. (2021). Cognitive biases, environmental, patient and personal factors associated with critical care decision making: A scoping review. *Journal of Critical Care*, 64, 144–153. 10.1016/j.jcrc.2021.04.012 33906103

[CIT0013] Braun, V., & Clarke, V. (2022). *Thematic analysis: A practical guide*. Sage.

[CIT0014] Brink, H., Van der Walt, C., & Van Rensburg, G. (2018). *Fundamentals of research methodology for healthcare professionals*. Juta.

[CIT0015] Bronfenbrenner, U. (1979). *The ecology of human development: Experiments by nature and design*. Harvard University Press.

[CIT0016] Campbell, M.L., Dove-Medows, E., Walch, J., Sanna-Gouin, K., & Colomba, S. (2011). The impact of a multidisciplinary educational intervention to reduce PEG tube placement in patients with terminal-stage dementia: A translation of research into practice. *Journal of Palliative Medicine*, 14(9), 1017–1021. 10.1089/jpm.2011.0041 21790469

[CIT0017] Car, L.T., El-Khatib, M., Perneczky, R., Papachristou, N., Atun, R., Rudan, I., Car, J., Vincent, C., & Majeed, A. (2017). Prioritizing problems in and solutions to homecare safety of people with dementia: Supporting carers, streamlining care. *BMC Geriatrics*, 17(1), 1–8. 10.1186/s12877-017-0415-6 28103810 PMC5244548

[CIT0018] Chou, H.H., Tsou, M.T., & Hwang, L.C. (2020). Nasogastric tube feeding versus assisted hand feeding in-home healthcare older adults with severe dementia in Taiwan: A prognosis comparison. *BMC Geriatrics*, 20(1), 1–8. 10.1186/s12877-020-1464-9 PMC7023686 32059646

[CIT0019] Cichero, J.A., Lam, P., Steele, C.M., Hanson, B., Chen, J., Dantas, R.O., Duivestein, J., Kayashita, J., Lecko, C., Murray, J., Pillay, M., Riquelme, L., & Stanschus, S. (2017). Development of international terminology and definitions for texture-modified foods and thickened fluids used in dysphagia management: The IDDSI framework. *Dysphagia*, 32(2), 293–314. 10.1007/s00455-016-9758-y 27913916 PMC5380696

[CIT0020] Cloete, M., Krüger, E., Van der Linde, J., Graham, M.A., & Pillay, S.B. (2022). South African speech-language therapists’ practices regarding feeding tube placement in people with advanced dementia. *South African Journal of Communication Disorders*, 69(1), 10. 10.4102/sajcd.v69i1.927 PMC9772722 36546518

[CIT0021] Colette, S., Langdon, K., & Bingham, M. (2022). The lived experience of hospice staff regarding the removal of artificial nutrition and hydration. *Palliative Medicine Reports*, 3(1), 199–206. 10.1177/26323222211007200

[CIT0022] De Jager, C.A., Msemburi, W., Pepper, K., & Combrinck, M.I. (2017). Dementia prevalence in a rural region of South Africa: A cross-sectional community study. *Journal of Alzheimer’s Disease*, 60(3), 1087–1096. 10.3233/JAD-170325 PMC5676974 28984589

[CIT0023] Desai, D.V., & Namasivayam-MacDonald, A.M. (2020). Speech-language pathologists’ perspectives on ethics in clinical practice. *American Journal of Speech-Language Pathology*, 29(2), 679–693. 10.1044/2019_AJSLP-19-00142

[CIT0024] Drenth, C., Sithole, Z., Pudule, E., Wüst, S., GunnClark, N., & Gwyther, L. (2018). Palliative care in South Africa. *Journal of Pain and Symptom Management*, 55(2), S170–S177. 10.1016/j.jpainsymman.2017.04.024 28803085

[CIT0025] Druml, C., Ballmer, P.E., Druml, W., Oehmichen, F., Shenkin, A., Singer, P., Soeters, P., Weimann, A., & Bischoff, S.C. (2016). ESPEN guideline on ethical aspects of artificial nutrition and hydration. *Clinical Nutrition*, 35(3), 545–556. 10.1016/j.clnu.2016.02.006 26923519

[CIT0026] Durepos, P., Wickson-Griffiths, A., Hazzan, A.A., Kaasalainen, S., Vastis, V., Battistella, L., & Papaioannou, A. (2017). Assessing palliative care content in dementia care guidelines: A systematic review. *Journal of Pain and Symptom Management*, 53(4), 804–813. 10.1016/j.jpainsymman.2016.10.368 28063859

[CIT0027] Dziewas, R., Baijens, L., Schindler, A., Verin, E., Michou, E., Clave, P., & European Society for Swallowing Disorders. (2017). European society for swallowing disorders FEES accreditation program for neurogenic and geriatric oropharyngeal dysphagia. *Dysphagia*, 32, 725–733. 10.1007/s00455-017-9828-9 28779300 PMC5674114

[CIT0028] Elliot, V., Morgan, D., Kosteniuk, J., Bayly, M., Froehlich Chow, A., Cammer, A., & O’Connell, M.E. (2021). Palliative and end-of-life care for people living with dementia in rural areas: A scoping review. *PLoS One*, 16(1), e0244976. 10.1371/journal.pone.0244976 33444351 PMC7808637

[CIT0029] Ellison, N. (2016). Supporting older people with dysphagia. *Journal of Dementia Care*, 24(2), 28–29.

[CIT0030] Espinosa-Val, M., Martín-Martínez, A., Graupera, M., Arias, O., Elvira, A., Cabré, M., Palomera, E., Bolivar-Prados, M., Clavé, P., & Ortega, O. (2020). Prevalence, risk factors, and complications of oropharyngeal dysphagia in older patients with dementia. *Nutrients*, 12(3), 863. 10.3390/nu12030863 32213845 PMC7146553

[CIT0031] Fong, J. (2019). Speech-language therapy for dysphagia management: Ethical and legal considerations in end-of-life care. *Perspectives of the ASHA Special Interest Groups*, 4(6), 1366–1378. 10.1044/2019_persp-19-00030

[CIT0032] Guastella, R., Oppedisano, S., Riquelme, L.F., & Namasivayam-MacDonald, A.M. (2022). Effects of cued and uncued swallowing in patients with dementia. *Perspectives of the ASHA Special Interest Groups*, 7(1), 139–148. 10.1044/2021_PERSP-21-00122

[CIT0033] Health Professions Council of South Africa (HPCSA). (2016). *Ethical guidelines for good practice in the health care professions*. Retrieved from https://www.hpcsa.co.za/Uploads/Professional_Practice/Ethics/Ethical_Guidelines_Booklet.pdf

[CIT0034] Health Professions Council of South Africa (HPCSA). (2019). *Guidelines for good practice in the healthcare professions: Dementia*. Retrieved from http://www.hpcsa.co.za/Uploads/editor/UserFiles/downloads/conduct_ethics/rules/generic_ethical_rules/booklet_14_guidelines_for_good_practice_in_dementia.pdf

[CIT0035] Hickey, E., & Bourgeois, M.S. (Eds.). (2017). *Dementia: Person-centered assessment and intervention*. Routledge.

[CIT0036] Huang, H.L., Lu, W.R., Liu, C.L., & Chang, H.J. (2020). Advance care planning information intervention for persons with mild dementia and their family caregivers: Impact on end-of-life care decision conflicts. *PLoS One*, 15(10), e0240684. 10.1371/journal.pone.0240684 33052970 PMC7556500

[CIT0037] Hwang, D., Teno, J.M., Gozalo, P., & Mitchell, S. (2014). Feeding tubes and health costs postinsertion in nursing home residents with advanced dementia. *Journal of Pain and Symptom Management*, 47(6), 1116–1120. 10.1016/j.jpainsymman.2013.08.007 24112820 PMC3979516

[CIT0038] Hydén, L.C., Majlesi, A.R., & Ekström, A. (2022). Assisted eating in late-stage dementia: Intercorporeal interaction. *Journal of Aging Studies*, 61, 101000. 10.1016/j.jaging.2022.101000 35654533

[CIT0039] Ijaopo, E.O., & Ijaopo, R.O. (2019). Tube feeding in individuals with advanced dementia: A review of its burdens and perceived benefits. *Journal of Aging Research*, 2019, 7272067. 10.1155/2019/7272067 31929906 PMC6942829

[CIT0040] Kochovska, S., Bugarski Ignjatovic, V., & Kozomara, R. (2020). Challenges and ethical dilemmas in end-of-life care for elderly persons with dementia. *Serbian Journal of Experimental and Clinical Research*, 21(1), 15–22. 10.2478/SJECR-2020-0002

[CIT0041] Kozlowski, D., Hutchinson, M., Hurley, J., Rowley, J., & Sutherland, J. (2017). The role of emotion in clinical decision making: An integrative literature review. *BMC Medical Education*, 17, 1–13. 10.1186/s12909-017-1089-7 29246213 PMC5732402

[CIT0042] Kuven, B.M. (2018). Interventions to increase oral intake in individuals with dementia: A systematic review. *Geriatric Nursing*, 39(4), 413–418. 10.1016/j.gerinurse.2018.02.007

[CIT0043] Luk, J.K., Chan, F.H., Hui, E., & Tse, C.Y. (2017). The feeding paradox in advanced dementia: A local perspective. *Hong Kong Medical Journal*, 23(3), 306–310. 10.12809/hkmj166110 28572521

[CIT0044] Malek, M.M., Abdul Rahman, N.N., Hasan, M.S., & Haji Abdullah, L. (2018). Islamic considerations on the application of patient’s autonomy in end-of-life decision. *Journal of Religion and Health*, 57(4), 1524–1537. 10.1007/s10943-018-0575-5 29417395

[CIT0045] Mash, R. (2016). Making sense of cultural safety, cultural humility and cultural competence in healthcare education. *African Journal of Primary Health Care & Family Medicine*, 8(2), 1–3. 10.4102/phcfm.v8i2.1259

[CIT0046] Meier, E.A., & Ong, M.S. (2015). A review of interventions to increase oral medication adherence in persons with cognitive impairment. *Journal of Gerontological Nursing*, 41(3), 20–29. 10.3928/00989134-26375146

[CIT0047] Mitchell, S.L. (2015). Advanced dementia. *New England Journal of Medicine*, 372(26), 2533–2540. 10.1056/NEJMcp1412652 26107053 PMC4539157

[CIT0048] Moser, A., & Korstjens, I. (2018). Series: Practical guidance to qualitative research. Part 3: Sampling, data collection and analysis. *European Journal of General Practice*, 24(1), 9–18. 10.1080/13814788.2017.1375091 29199486 PMC5774281

[CIT0049] National Institute for Health and Clinical Excellence (NICE). (2006). *Nutrition support in adults: Oral nutrition support, enteral tube feeding and parenteral nutrition*. Clinical guideline CG32. Retrieved from https://www.nice.org.uk/guidance/cg32/resources/nutrition-support-in-adults-oral-nutrition-support-enteral-tube-feeding-and-parenteral-nutrition-pdf-975631858053 31999417

[CIT0050] National Policy Framework and Strategy for Palliative Care. (2017). *South African government*. Retrieved from https://www.gov.za/sites/default/files/gcis_document/201711/41277gon1267.pdf

[CIT0051] Newman, R.D., Ray, R., Woodward, L., & Glass, B. (2019). Factors contributing to the preferred method of feeding in end-stage dementia: A scoping review. *Dysphagia*, 35, 1–14. 10.1007/s00455-019-10072-3 31616996

[CIT0052] Panther, K. (2005). The Frazier free water protocol. *Perspectives on Swallowing and Swallowing Disorders (Dysphagia)*, 14(1), 4–9. 10.1044/sasd14.1.4

[CIT0053] Payne, C., & Morley, J. (2018). End of life care in advanced dementia: A review of the literature. *Dementia (London, England)*, 17(1), 43–60. 10.1177/1471301216672129

[CIT0054] Punchik, B., Shinan-Altman, S., & Golander, H. (2018). Food and drink consumption by people with dementia in long-term care facilities: A systematic review. *Journal of Advanced Nursing*, 74(12), 2744–2756. 10.1111/jan.13817

[CIT0055] Schwartz, D.B. (2018). Enteral nutrition and dementia integrating ethics. *Nutrition in Clinical Practice*, 33(3), 377–387. 10.1002/ncp.10085 29665095

[CIT0056] Schwartz, R.K., Szydlowski, J., & Day, A. (2014). Oral intake in persons with advanced dementia: A difficult problem with no easy solutions. *Journal of Geriatric Psychiatry and Neurology*, 27(2), 112–119. 10.1177/0891988714525412

[CIT0057] Standards for Palliative Care in South Africa. (2017). *Hospice palliative care association of South Africa*. Retrieved from https://hpca.co.za/wp-content/uploads/2017/06/2017-Standards-for-Palliative-Care-in-SA.pdf

[CIT0058] Stellenberg, E.L., & De Wet, N.C. (2016). Cultural competence of speech-language pathologists in South Africa: Perceptions of university educators. *South African Journal of Communication Disorders*, 63(1), e1–e8. 10.4102/sajcd.v63i1.143

[CIT0059] Terry, G., Hayfield, N., Clarke, V., & Braun, V. (2017). Thematic analysis. In B. Taylor (Eds.), *The SAGE handbook of qualitative research in psychology*, Vol. 2 (pp. 17–37). SAGE Publications.

[CIT0060] Tsao, J.C., Fletcher, B.R., Hartnoll, R., & Craven, H. (2019). Promoting end-of-life discussions and advance care planning in speech and language therapy. *International Journal of Palliative Nursing*, 25(2), 86–93. 10.12968/ijpn.2019.25.2.86

[CIT0061] Varindani Desai, R., & Namasivayam-MacDonald, A. (2020). Practice patterns of speech-language pathologists managing dysphagia in dementia: A cross-sectional survey in the United States. *Perspectives of the ASHA Special Interest Groups*, 5(6), 1631–1646. 10.1044/2020_PERSP-19-00152

[CIT0062] Volicer, L., Pope, T.M., & Steinberg, K.E. (2019). Assistance with eating and drinking only when requested can prevent living with advanced dementia. *Journal of the American Medical Directors Association*, 20(11), 1353–1355. 10.1016/j.jamda.2019.08.035 31676025

[CIT0063] Volkert, D., Chourdakis, M., Faxen-Irving, G., Frühwald, T., Landi, F., Suominen, M.H., Vandewoude, M., Wirth, R., & Schneider, S.M. (2015). ESPEN guidelines on nutrition in dementia. *Clinical Nutrition*, 34(6), 1052–1073. 10.1016/j.clnu.2015.09.004 26522922

[CIT0064] Vose, A.K., Kesneck, S., Sunday, K., Plowman, E., & Humbert, I. (2018). A survey of clinician decision making when identifying swallowing impairments and determining treatment. *Journal of Speech Language and Hearing Research*, 61(11), 2735. 10.1044/2018_JSLHR-S-17-0212 PMC7242916 30458527

[CIT0065] Wirth, R., Dziewas, R., Beck, A.M., Clavé, P., Hamdy, S., Heppner, H.J., Langmore, S., Leischker, A., Martino, R., Pluschinski, P., Rösler, A., Shaker, R., Warnecke, T., Sieber, C., & Volkert, D. (2016). Oropharyngeal dysphagia in older persons–from pathophysiology to adequate intervention: A review and summary of an international expert meeting. *Clinical Interventions in Aging*, 11, 189–208. 10.2147/CIA.S97481 26966356 PMC4770066

[CIT0066] Wright, C., Duffield, C., & Hennessey, C. (2019). Palliative care for people with dementia living in the community: A systematic review and meta-analysis. *Palliative Medicine*, 33(6), 724–736. https://doi:10.1177/0269216319845347

[CIT0067] Yuen, J.K., Luk, J.K., Chan, T.C., Shea, Y.F., Chu, S.T., Bernacki, R., Chow, D.T.Y., & Chan, F.H.W. (2022). Reduced pneumonia risk in advanced dementia patients on careful hand feeding compared with nasogastric tube feeding. *Journal of the American Medical Directors Association*, 23(9), 1541–1547. 10.1016/j.jamda.2022.03.011 35489380

